# Reducing the Wire Crossing Time in Primary Percutaneous Coronary Angioplasty: A Study From a Tier II City in India

**DOI:** 10.7759/cureus.21539

**Published:** 2022-01-24

**Authors:** Binayendu Prakash, Reeta R Mohanta, Prem P Lal, Mandar M Shah

**Affiliations:** 1 Department of Cardiology, Tata Main Hospital, Jamshedpur, IND

**Keywords:** time delay, cathlab, st-elevation myocardial infarction (stemi), primary percutaneous coronary intervention (pci), wire crossing time

## Abstract

Acute coronary syndrome is a major cause of morbidity and mortality all over the world. Timely intervention in ST-elevation myocardial infarction (STEMI) in the form of primary angioplasty is the gold standard of treatment to reduce mortality and morbidity. “Time is muscle” is the phrase to impress upon the importance of time in treating patients with STEMI. Traditional treatment target included “door to balloon time” of 90 min or less. This “door to balloon time” is now rephrased as the “wire crossing time” (WCT). The European Society of Cardiology (ESC) updated its guidelines further, reducing the target of wire crossing time to 60 min.

The present study is a brief report on the door to wire crossing time status in one of the tertiary care centers of a nonmetro city. Retrospective analysis of case records was done for 79 patients admitted with acute MI who underwent primary angioplasty between November 2018 and June 2019 (pre-corrective action group). Various reasons for the delay, right from the time of the patient reaching the emergency room (ER) to the time of wire crossing, were analysed and measures were taken to reduce the delay. The post-corrective action group comprised 77 patients. The major causes of a prolonged WCT in our setup were delayed diagnosis of STEMI in ER, delay in giving consent by the patient’s relatives, financial issues, and availability of cath lab technicians during the off-duty hour. The delay in WCT in our center was 121 min. Remedial actions were taken to mitigate the problems at each step, which resulted in a reduction of delay by 20 min, i.e., to 101 min leading to a significant difference in the outcome in view of morbidity and mortality.

## Introduction

Acute coronary syndrome is a major cause of morbidity and mortality all over the world. Timely intervention in ST-elevation myocardial infarction (STEMI) is one of the major successes in interventional cardiology. The preferred treatment for patients presenting with STEMI is primary percutaneous coronary intervention (PCI) [1]. There cannot be a better phrase than “time is muscle” to impress upon the importance of time in treating patients with STEMI [2]. Several studies have found a significant relationship between prolonged ischemic times and poor outcomes [3-6]. Delays in reperfusion negatively influence mortality and morbidity.

The critical determinant of outcome is time to treatment [7]. There are many related parameters that have been studied to measure the time lost in STEMI management, such as symptom-to-door (S2D), door-to-door, and door-to-device (D2D) times. The D2D time, as well as the more recently modified measure, first-medical-contact to the device, has been suggested as an indicator of the quality of care in STEMI management [8]. Traditional treatment targets include a “door to balloon time” of 90 min or less, with updated guidelines of a 60 min target from taking ECG to vessel opening (wire crossing time [WCT]) cited in the most recent European Society of Cardiology (ESC) guidelines [9].

In the present study, the door to wire crossing time was studied in patients with STEMI, who underwent primary percutaneous coronary angioplasty in the Tata Main Hospital (TMH), Jamshedpur, a tier II city of India. The contributing factors to the prolonged WCT were analysed and measures were taken to reduce it.

## Materials and methods

TMH is a 980-bedded multispeciality, tertiary care hospital catering to Tata Steel company employees and their dependents as well as the non-employee general population of the city of Jamshedpur and surroundings.

The Department of Cardiology, TMH, is equipped with one cath lab, 22-bedded intensive care unit, manned 24 hours by doctors on duty (DODs). Three interventional cardiologists are available for intervention during office hours and on-call during off-duty hours. There are three cath lab technicians to run the equipment. They are also available on duty during office hours and on call during off-duty hours.

Patients come to the general emergency room (ER), which is manned round the clock by ER DODs and physicians. On arriving in the ER, the DOD takes a detailed history of the patient and ECG is recorded. If ECG reveals STEMI, cath lab is alerted and the patient is shifted there. On reaching the cath lab, cardiology DOD reviews the patient and ECG, and assesses patient’s suitability for primary angioplasty. He or she then informs the interventional cardiologist, and discusses the need of primary angioplasty with the patient’s relatives, along with the pros, cons, and financial implications. The patient is taken for the procedure if consent is given. During off-duty hours, the cardiologist and cath lab technicians are called from home.

It is to be noted that primary angioplasty is done free of cost for active employees of the company and their dependents. Associate company employees need to obtain a consent letter from their company; till then, permission from their company is obtained on phone. Ex-employee and general non-employee patients need to pay full charges before the onset of the procedure.

This was a two-phase study. The first phase was a retrospective study of patients with STEMI who underwent primary PCI in TMH between November 2018 and June 2019. In the case of a prolonged WCT, the patient’s medical records were examined to find the causes of the delay. Patients with STEMI who were not a candidate for primary PCI for any reason were excluded, such as those having prohibitive comorbid conditions, refusing to undergo primary PCI, and death before PCI. A preliminary analysis was conducted to identify the major causes of prolonged time delay. The time delay in each step was analysed by starting a self-initiated project and corrective measures were taken to reduce time delay.

The second phase, a prospective study, commenced in July 2019 to check the effect of remedial measures. Further data were collected from July 2019 to March 2020 and were compared with the previous data to check for improvement.

The study was presented to the Tata Main Hospital Institutional Ethics Committee (TMH IEC) on December 20, 2021, and approved for publication (TMH/IEC/DEC/071/2021).

## Results

Various reasons for delay, right from the time of the patient reaching the ER to the time of wire crossing, were evaluated. They were clubbed into two broad groups A and B and further classified into various subcategories; possible remedial measures were taken to rectify them (Tables 1, 2).

**Table 1:** Delay from the time of reaching the ER to shifting in the cath lab (group A) ER, emergency room; STEMI, ST-elevation myocardial infarction

	Subcategories	
1.	Delay in taking history/ECG	
	Reasons/hurdles: The emergency being over-crowded on most the days, and catering to a variety of patients including non-cardiac patients; often, there was a delay in attending to the patient, taking proper history and getting an ECG done in patients with chest pain.	Remedial actions taken: Discussion with ER doctors to expedite ECG in all patients presenting with typical symptoms.
2.	Delay in interpreting ECG and review by the ER physician	
	Reasons/hurdles: The ER is primarily manned by MBBS-level doctors who, when unsure of the diagnosis, would seek the opinion of a physician on call, and then the physician on call would interpret the ECGs. This often resulted in unwarranted delay.	Remedial actions taken: Soft copy of ECG to be shared with the physician and the cardiologist for quick reference.
3.	Availability of shifting logistics	
	Reasons/hurdles: There were issues noted on some occasions such as unavailability of patient trolleys or ward attendants since the attendants had already gone to shift some other patients to other wards. Also, it is noteworthy that the distance between the ER and cath lab ward is over 300 meters and usually takes around 7-8 min to reach.	Remedial actions taken: Ensured adequate number of shifting persons and trolleys all the time.
4.	Availability of beds in the cath lab	
	Reasons/hurdles: On many occasions, the cath lab beds are fully preoccupied. Once the cath lab ward receives information from the ER regarding acute STEMI patient in the ER, it would take time to mobilize an admitted patient from the cath lab ward to the other ward to make bed available for the new patient.	Remedial actions taken: Always to keep one bed available for STEMI; following the “never say no to STEMI” policy.

**Table 2:** Delay between reaching the cath lab and wire crossing (group B) ER, emergency room; PCI, percutaneous coronary intervention

	Subcategories	
1.	Counselling the patient’s relatives about the procedure	
	Reasons/hurdles: Occasionally, patients are brought by neighbors or colleagues, and decision-makers of the family are not available. This leads to unnecessary delays.	Remedial actions taken: Counselling to be done from the ER itself.
2.	Relatives giving consent	
	Reasons/hurdles: There are multiple factors resulting in the delay by the relatives in giving consent: disbelief and denial of the diagnosis (“patient was completely normal till a short while ago”), urge to get a second opinion, inability to arrange finances at a short notice, among others.	Remedial actions taken: Counselling about the need of early intervention done.
3.	During off-duty office hours - calling the cardiologist and technicians	
	Reasons/hurdles: Although the cardiologists stay within the vicinity of the hospital and can reach within a few minutes, the technicians stay at the peripheries. Some of them don’t even have a personal vehicle and need a hospital vehicle to be sent to their house to fetch them, thus resulting in undue delays.	Remedial actions taken: Employing one extra technician to make possible 24-hour availability.
4.	Financial	
	Reasons/hurdles: The active employees of the Tata Steel company and their family members are entitled to free treatment up to 2 stents. However, others are required to make a full payment. For an expensive procedure like PCI, relatives often cannot make up their minds at a short notice. Also, even if they are convinced and willing, arranging funds at such a short notice often proves to be a challenge. Also, the mode of payment being accepted was card/demand draft/cash. For most card payments, there is a set limit for daily transactions.	Remedial actions taken: All modes of payment to be accepted.

Furthermore, prospective data were collected to check the effect of corrective measures. The pre- and post-corrective data were analysed. Means and standard deviations were calculated for continuous variables, and frequencies and percentages were calculated for discrete variables. An independent t-test was used to compare the two groups and find its significance value. The chi-square test was used to compare the discrete variables in two groups.

Retrospective analysis of the case sheet was done for 79 patients admitted with acute STEMI who underwent primary angioplasty between November 2018 and June 2019 (pre-corrective action group). The mean age was 58 years; 63 were males and 16 were females. The post-corrective action group comprised 77 patients with a mean age of 59 years and had 66 males and 11 females. The demographic data of both the groups were comparable with no significant difference in age (p-value 0.65) and sex (p-value 0.32) (Tables 3, 4).

**Table 3:** Demographic data of patients

	Male	Female	p-value
Pre-corrective measure group (N=79)	63	16	0.325
Post-corrective measure group (N=77)	66	11	

**Table 4:** Demographic data, based on age

Variable	Pre-corrective measure group	Post-corrective measure group	p-value
Age (years)	58.96 ± 11.49	59.87 ± 13.65	0.65

The time delay from the ER to cath lab was 34 min, which got reduced after corrective measures to 30 min, which was not significant (p-value 0.56), but the delay from reaching the cath lab to wire crossing was significantly reduced from 86 min to 71 min after corrective measures (p-value 0.034). Overall, there was a significant reduction in the total time delay from 121 min to 101 min (p-value 0.039) (Table 5).

**Table 5:** Comprehensive data

Variable	Pre-corrective measures (N=79)	Post-corrective measures (N=77)	p-value
Age (years)	58.96 ± 11.49	59.87 ± 13.65	0.65
ER to cath lab (min)	34.04 ± 37.27	30.79 ± 41.38	0.560
Cath lab to wire crossing (min)	86.87 ± 52.62	71.23 ± 37.29	0.034
Total time delay (min)	121 ± 61.98	101.59 ± 2.08	0.039

Subgroup analysis

Patients were further grouped into two categories: paying and non-paying patients. The non-paying group comprised employees, employee family, employee recovery, and associate company patients. The paying group comprised non-employees, ex-employees, and their family members.

Non-Paying Patients

In the non-paying group, there were 30 patients. Before corrective measures, ER delay was 39.9 min and cath lab delay was 78.9 min. The total delay recorded was 118.9 min. After corrective measures, the total patients studied were 22; ER delay was reduced to 22 min and cath lab delay was reduced to 62 min. Thus, the total delay was reduced to 84 min, which was a significant reduction (p-value 0.002) (Table 6).

**Table 6:** Pre- and post-corrective measure comparison in non-paying patients

Variable	Pre-corrective measures (N=30)	Post-corrective measures (N=22)	p-value
Age (years)	60.66 ± 9.57	62.61 ± 12.56	0.53
ER to cath lab (min)	39.91 ± 44.15	19.95 ± 10.40	0.043
Cath lab to wire crossing (min)	79.59 ± 40.54	62.05 ± 28.87	0.090
Total time delay (min)	119.48 ± 51.81	81.91 ± 10.40	0.002

Paying Patients

In the paying group, there were 49 patients. Before corrective measures, the ER delay was 30 min and cath lab delay was 91 min. The total delay recorded was 122 min. After taking corrective measures, the total patients studied were 55; ER delay was 35 min and cath lab delay reduced to 74 min. The total delay reduced to 109 min, which was not significant (p-value 0.253) (Table 7).

**Table 7:** Pre- and post-corrective measure comparison in paying patients

Variable	Pre-corrective measures (N=49)	Post-corrective measures (N=55)	p-value
Age (years)	57.98 ± 12.46	58.82 ± 14.01	0.72
ER to cath lab (min)	30.64 ± 32.64	34.56 ± 47.86	0.580
Cath lab to wire crossing (min)	91.1 ± 58.47	74.91 ± 39.81	0.078
Total time delay (min)	122.96 ± 67.65	109.47 ± 65.16	0.253

The overall delay in the door to wire crossing time in our center was 121 min, while the expected delay is supposed to be less than 60 min, as per the latest ESC guidelines.

## Discussion

In developed countries, many studies have been conducted on the door-to-door time or door to balloon time. There was a continuous decline in the door-to-door time in the United States from 111 min in 1994 to 79 min in 2006 [10] and further to 64 min in 2010 [11]. The door-to-door time was reported to be 64 min in the Netherlands in 2012 [12], 92 min in Japan in 2013 [13], and 65 min in Australia in 2014 [14].

India, being a developing country with limited resources and infrastructure, has very few cardiac-specific hospitals and improving cardiac emergency in a multidisciplinary hospital still remains a challenge. The door-to-door time was reported to be 75 min in a single center [15]. A study from Kazakhstan showed that the door-to-door time decreased from 155 min to 73 min over a time period between 2012 and 2015 [16].

There are several suggestive measures to reduce the door-to-door time and centers should choose one or more based on local considerations. Some of these strategies require investment in infrastructure such as providing prehospital ECGs, while some others can be applied in most hospitals, with limited budgets, by modifying the internal protocols such as the activation of the STEMI code by emergency physicians and the direct transfer of patients to the cath lab, bypassing the ER [17-21].

The present study is a brief report on the door to wire crossing time status in one of the multispeciality hospitals of a tier II city in a developing country, India. The delays were reduced by implementing improvement measures. Actions were taken to mitigate the problems at each step, which resulted in a reduction in delay by 20 min, i.e., 16.53%, which made a significant difference in the outcome in view of morbidity and mortality.

One of the major causes of the prolonged wire crossing time in our setup was delayed diagnosis of STEMI in ER. Hence, the importance of time in managing STEMI was reinforced to ER doctors. Also, a better collaboration between ER doctors and cardiologists was established. An adequate number of transfer trolleys and personnel was ensured to reduce the delay in the transfer of a patient to the cath lab. A policy of “never say no to STEMI patient” was adopted and one bed was always reserved for such patients. Extra cath lab technicians were employed to manage cath lab emergencies round the clock.

Subgroup analysis showed a significant reduction in time delay in the non-paying group, where as a reduction in time delay was not significant in the paying group, indicating financial issues. Limited medical insurance coverage often leads to a delay in making an important decision about primary angioplasty, more so during non-banking hours. Financial issues and informed consent were reported as two major causes of delay in one of the studies from India [15].

Our study showed that even in a high-volume multispeciality center, continuous monitoring of the wire crossing time and improvement in the processes can significantly reduce the time delay.

## Conclusions

Acute coronary syndrome is a major cause of morbidity and mortality all over the world. The time-tested metric of the door to wire crossing time remains an important quality parameter in the management of primary percutaneous coronary angioplasty. Several measures have been tested over time to reduce the WCT, leading to reduced morbidity and mortality.

While our WCT was reduced to 101 min, yet it was above the international target of 60 min. Although it is difficult to achieve the international target, yet it is possible to reduce it significantly by identifying the reasons of delay and taking remedial measures at each step. Giving preference to patients with chest pain, quickly recording ECG to diagnose STEMI, patient counselling, alerting the cath lab, and speedy transfer can expedite the processes. Upgrading the healthcare sector by adding more number of cardiac-specific hospitals and providing a wider medical insurance coverage to the general population can remarkably reduce delays in primary angioplasty, thereby improving the cardiac care.
